# AI literacy and foreign language boredom in AI-assisted academic English learning: the roles of engagement and task value

**DOI:** 10.3389/fpsyg.2026.1795301

**Published:** 2026-04-13

**Authors:** Fang Liu

**Affiliations:** College of Foreign Languages, Guilin University of Electronic Technology, Guilin, Guangxi, China

**Keywords:** academic pressure, AI literacy, Chinese EFL postgraduates, foreign language boredom, learner engagement, task value

## Abstract

With the rapid integration of artificial intelligence (AI) tools into academic English learning, understanding how learners’ AI-related competencies influence their motivational and emotional experiences has become increasingly important. Drawing on frameworks of technology enhanced learning and foreign language emotions, this study examines the relationship between AI literacy and foreign language boredom in AI-assisted academic English learning, with a particular focus on the roles of learner engagement and task value. Survey data were collected from 392 postgraduate students and analyzed using Exploratory Factor Analysis and Structural Equation Modeling. The results indicated that AI literacy played a foundational role by positively associating with both learning engagement and perceived task value. Learning engagement was found to reduce levels of foreign language boredom and academic pressure, whereas academic pressure emerged as a strong positive predictor of foreign language boredom. Interestingly, perceived task value showed a positive association with boredom, suggesting that highly valued academic English tasks may simultaneously evoke emotional fatigue in high demand learning environments. There is no moderating effect of perceived task value on the relationship between engagement and boredom. Overall, the findings highlight the complex interplay between AI-related competencies, motivational processes, and emotional experiences in AI-assisted foreign language learning. The study underscores the importance of fostering AI literacy and sustained learning engagement while also addressing academic pressure to promote emotionally sustainable AI-supported language learning environments.

## Introduction

With the rapid integration of artificial intelligence (AI) technologies into higher education, AI-assisted academic English learning has become increasingly prevalent, particularly among students who face growing demands for academic reading, writing, and publication in English (Biju et al., 2024; Song and Song, 2023). Tools such as AI-based writing assistants, automated feedback systems, and large language models are now widely used to support academic English learning by enhancing efficiency, accessibility, and individualized support (AbuSahyon et al., 2023; La De Vall and Araya, 2023; Son et al., 2025). While prior research has largely emphasized the cognitive and performance-related benefits of AI-assisted language learning (Xu et al., 2025), considerably less attention has been paid to learners’ affective experiences in these technology-rich environments, especially negative emotions such as foreign language boredom (Wang, 2025).

Foreign language boredom has recently emerged as a critical yet underexplored emotion in second and foreign language learning research (Li, 2025; Li et al., 2023). Conceptualized as a low-arousal, negative achievement emotion characterized by disengagement, monotony, and attentional difficulties, boredom has been shown to undermine learners’ motivation, persistence, and learning outcomes (Coşkun and Yüksel, 2022; Van Tilburg and Igou, 2017). Compared with anxiety and enjoyment, boredom has received relatively limited empirical attention, until recent years, when it began to attract growing scholarly interest as a distinct emotional experience in second language learning (Dewaele et al., 2023a, 2023b; Liu and Cho, 2025; Pawlak et al., 2020).

Although foreign language boredom has been examined in traditional classroom settings, its characteristics in AI-assisted learning environments remain underexplored. In conventional classrooms, boredom is often associated with repetitive instructional practices, limited task variety, overreliance on automated tools, reduced cognitive challenge, limited meaningful interaction, or teacher-centered teaching approaches (Dewaele et al., 2023a, 2023b; Li, 2025; Li et al., 2023; Liu and Cho, 2025; Pawlak et al., 2020). In contrast, AI-assisted learning may introduce different triggers of boredom, such as a lack of technological proficiency (Jones and Graham, 2020), passive interaction with AI, the overreliance on automated AI outputs, cognitive overload from AI tools, reduced cognitive effort when interacting with generative tools, or uncertainty about how to critically evaluate AI-generated content (Fryer et al., 2020; Pérez et al., 2020; Wu, 2025). These contextual differences suggest that foreign language boredom in AI-assisted learning environments may involve distinct mechanisms and influencing factors, highlighting the need to investigate how technological competence and motivational processes shape learners’ emotional experiences in AI-supported language learning.

At the same time, learners’ ability to effectively and critically use AI technologies was commonly conceptualized as AI literacy (Ng et al., 2021). AI literacy has become a key individual difference shaping learning experiences in AI-enhanced environments (Yi, 2021). AI literacy generally refers to learners’ knowledge of AI functionalities and limitations, their skills in using AI tools appropriately, and their capacity to evaluate, reflect on, and regulate AI-supported learning processes (Chiu et al., 2024). Existing studies suggest that higher levels of AI literacy are associated with greater learner autonomy, more strategic technology use, and improved learning engagement (Lee et al., 2021). However, empirical evidence remains scarce regarding how AI literacy relates to learners’ emotional experiences, particularly negative emotions such as boredom, in academic English learning contexts.

From a motivational and emotional perspective, learner engagement and task value are two theoretically relevant mechanisms through which AI literacy may influence foreign language boredom. Learner engagement, encompassing behavioral, cognitive, and emotional involvement in learning activities, has consistently been identified as a key protective factor against boredom (Bekker et al., 2023). Learners who are more actively engaged are less likely to experience attentional drift, monotony, or emotional withdrawal (Derakhshan et al., 2024; Liu et al., 2022). In AI-assisted learning environments, AI literacy may enable learners to interact with AI tools more purposefully, thereby fostering deeper engagement and reducing boredom (Saleh and Alsubhi, 2025).

Task value, defined as learners’ perceptions of the usefulness, importance, and meaningfulness of learning tasks, also plays a central role in shaping achievement emotions. Task value and academic self-efficacy affect boredom and attention in class (Sánchez-Rosas and Esquivel, 2016), and task value was positively related to enjoyment and negatively related to boredom (Noteborn et al., 2012). According to expectancy–value theory, tasks perceived as valuable are more likely to elicit positive emotions and sustained effort, whereas low task value is closely associated with boredom and disengagement (Cui et al., 2017). In academic English learning, especially at the postgraduate level, AI-literate learners may better recognize how AI-assisted tasks contribute to their academic goals, such as thesis writing or research publication, thereby enhancing task value and potentially mitigating boredom.

In addition, academic pressure is a salient contextual factor for postgraduate EFL learners, who often face high expectations related to English proficiency, publication requirements, and academic performance. Prior research has shown that excessive academic pressure may intensify negative emotions, including anxiety, burnout, depression and boredom, particularly when learners lack sufficient coping resources (Bhujade, 2017; Dimitrov, 2017; Kumaraswamy, 2013). Engagement has been identified as a potential buffer against academic pressure (Liu, 2024), yet the interplay among engagement, pressure, and boredom within AI-assisted language learning remains insufficiently understood.

Despite the growing body of research on AI in language education, several gaps remain. First, empirical studies examining foreign language boredom in AI-assisted academic English learning contexts are still limited. Second, little is known about the role of AI literacy in shaping learners’ emotional experiences beyond performance outcomes. Third, the underlying pathways linking AI literacy to boredom, particularly the mediating roles of engagement and task value and the involvement of academic pressure, have not been systematically examined using robust analytical approaches.

## Objectives of the study

The present study aims to examine the structural relationships among AI literacy, learner engagement, task value, academic pressure, and foreign language boredom in the context of AI-assisted academic English learning among Chinese EFL postgraduates. Specifically, the objectives are as follows:

1. To investigate the extent to which AI literacy associates with learner engagement and perceived task value in AI-assisted academic English learning.
2. To examine the associations between learner engagement, task value, and foreign language boredom, with a particular focus on their emotional–motivational implications.
3. To determine the role of academic pressure in explaining foreign language boredom among postgraduate EFL learners.
4. To explore whether learner engagement is associated with reduced academic pressure in academically demanding EFL contexts.
5. To test the mediating roles of learner engagement and task value in the relationship between AI literacy and foreign language boredom.
6. To further examine whether academic pressure serves as an explanatory pathways linking learner engagement to foreign language boredom.

## Literature review and theoretical framework

### AI literacy in AI-assisted academic English learning

The rapid integration of artificial intelligence (AI) into higher education has reshaped the landscape of academic English learning (Zawacki-Richter et al., 2019), particularly for postgraduate EFL learners who increasingly rely on AI tools for reading, writing, and research-related tasks (Song and Song, 2023; Wei, 2023). Within this context, AI literacy has emerged as a critical learner competence (Long and Magerko, 2020). According to Ng et al. (2021), AI literacy generally refers to individuals’ ability to understand the capabilities and limitations of AI systems, critically evaluate AI-generated outputs, and strategically employ AI tools in ethically responsible ways to support learning.

Recent studies have begun to conceptualize AI literacy as an individual difference variable that extends beyond technical proficiency to include metacognitive awareness and critical judgment. In language learning contexts, AI literacy has been shown to influence learners’ self-efficacy, perceived control, and affective responses when interacting with AI-assisted tasks (Fan and Zhang, 2024; Zhang et al., 2025). Empirical evidence from EFL settings suggests that learners with higher AI literacy tend to report greater self-efficacy, stronger learning engagement (Wang et al., 2025), more willingness to communicate (Liu and Fan, 2025) and lower levels of negative emotions such as anxiety (Zhang et al., 2025), or boredom (Wu and Xu, 2025), particularly when AI tools are used for complex academic purposes such as writing and literature review tasks (Jalambo et al., 2025; Rad et al., 2024; Wang and Wang, 2025). Namaziandost (2025) stated that the study showed a substantial reduction in foreign language learning boredom for learners engaged in the AI-enhanced flipped classroom. Similarly, EFL learners reported lower boredom, reduced anxiety and satisfaction with AI chatbots (Jalambo et al., 2025). A study among 465 EFL students in China showed a positive effect of adopting AI in enhancing foreign language enjoyment and engagement and reducing foreign language anxiety and boredom compared to the traditional EFL classroom setting (Dong et al., 2026). Complex interaction between foreign language emotions and engagement in AI-assisted EFL classrooms is revealed (Dong et al., 2026; Jin and Wu, 2026). These findings indicate that AI literacy may play a foundational role in shaping learners’ motivational and emotional experiences in AI-assisted academic English learning environments (Liu and Fan, 2025; Wang, 2025).

### Engagement and perceived task value in AI-assisted language learning

Learner engagement has long been recognized as a key determinant of successful second language acquisition (Hiver et al., 2024). Engagement is typically conceptualized as a multidimensional construct encompassing behavioral involvement, cognitive effort, and emotional investment in learning activities (Baralt et al., 2016; Fredricks et al., 2004; Henry and Thorsen, 2020; Lambert et al., 2017). In AI-assisted learning environments, engagement is often facilitated through features such as immediate feedback, adaptive scaffolding, and interactive task design (Jafarian and Kramer, 2025; Nguyen et al., 2024). Mixed study by Huang (2025) revealed significant correlations between AI-assisted language learning engagement and students’ language outcomes among Chinese EFL students. However, learners’ engagement is not solely driven by technological affordances (Liu, 2025); it is also contingent on learners’ competencies and perceptions when interacting with AI systems.

From a motivational perspective, perceived task value, defined as learners’ beliefs about the usefulness, importance, and relevance of learning tasks, plays a central role in sustaining engagement (Eccles and Wigfield, 2002; Wigfield and Eccles, 1992). For instance, Chiu and Wang (2008) concluded that attainment value, utility value, and intrinsic value were significant predictors of individuals’ intentions to continue using Web-based learning. Marchand and Gutierrez (2017) reported midsemester value belief predicted student reports of behavioral and cognitive engagement. Xie et al. (2024) studied and found typical non-English major students were less interested in the L2 learning task. In addition, task value determined the persistence and demotivation, strongly “tied to achievement-related choices” (p.41). Drawing on expectancy–value theory, prior research consistently demonstrates that learners who perceive higher task value are more likely to invest effort, persist in challenging tasks, and experience more adaptive emotional states. Liem et al. (2008) found task value predicted mastery goals of EFL students. Similarly, according to Johnson and Sinatra (2013), task value induction demonstrated higher engagement and conceptual change. In academic English learning, especially at the postgraduate level, perceived task value is closely tied to learners’ academic and professional goals, such as thesis writing, publication, and future career development.

Emerging research in AI-assisted EFL contexts suggests that learners’ perceptions of task value may be influenced by how effectively AI tools support meaningful academic outcomes. When learners perceive AI-assisted tasks as instrumental for improving academic English competence, engagement is likely to increase (Huang, 2025). Conversely, when AI use is perceived as superficial, repetitive, or misaligned with academic goals, engagement may diminish, potentially leading to negative emotional experiences.

### Foreign language boredom and academic pressure

In recent years, research on second language emotions has expanded beyond anxiety to include foreign language boredom, a low-arousal negative emotion characterized by disengagement, attentional difficulties, and a perceived lack of stimulation or meaning (Kruk et al., 2022; Li et al., 2023; Pawlak et al., 2021). Empirical studies have shown that boredom is prevalent in language classrooms and is associated with reduced engagement, lower motivation, and poorer learning outcomes (Dewaele et al., 2023a, 2023b; Li et al., 2023). In contrast to anxiety, which is often linked to high cognitive demands, boredom is more closely associated with under-stimulation, repetitive tasks, or low perceived value (Pawlak et al., 2025). Based on control-value theory, Li (2021) conceptualized and validated a 7-factor Foreign Language Learning Boredom Scale. A recent study continued to structure teacher-related factors on foreign language boredom (Liu and Cho, 2025). Though great focus has been put on antecedents and effects of foreign language boredom, scarce explorations have been made in terms of instructional intervention to mitigate this negative feeling (Zhang and Li, 2025).

Recent studies suggest that contextual pressures within academic environments may further shape learners’ boredom experiences. In postgraduate academic English learning, academic pressure represents an additional contextual factor that may exacerbate negative emotional experiences and educational achievement (Hendra et al., 2025). Task overload was identified as one of the main stressors which might lead to burnout, exhaustion and negative psychological consequences (Pérez-Jorge et al., 2025; Xu et al., 2023). High-stake assessment was frequently reported as school-related distress among high school students (Jagiello et al., 2025). Thus, high academic demands related to research productivity, publication expectations, and time constraints can heighten stress and emotional fatigue. When learners experience high pressure without sufficient perceived control or value in learning tasks, boredom may paradoxically coexist with stress, reflecting emotional disengagement under demanding conditions (Klimova and Pikhart, 2025).

### The role of academic pressure in language learning emotion research

Academic emotions among graduate students are largely shaped by academic pressure arising from performance demands, interpersonal expectations, and career-related concerns (Xu et al., 2023). Empirical evidence consistently shows that academic workload, examinations, and performance expectations constitute major stressors in higher education contexts. For instance, Ahuja et al. (2022) reported that a large majority of graduate students experienced moderate to high levels of academic stress, with pressure to perform emerging as the primary stress source.

Within second language learning contexts, academic pressure has increasingly been linked to learners’ motivational and emotional experiences. Liang and Mao (2025) found that academic stress negatively predicted academic motivation among Chinese L2 learners, particularly under heavy workload and examination pressure. Similarly, Alqarni (2024) demonstrated that perceived academic stress interacts with emotion regulation to influence EFL proficiency, suggesting a dynamic relationship between academic demands and learners’ emotional experiences.

Recent research further indicates that the emotional impact of academic pressure may vary across individual characteristics and psychological resources. Gender differences have been shown to persist in language-related anxiety even when psychological variables are controlled (Huerta et al., 2017). In addition, higher emotional intelligence has been associated with lower stress and burnout as well as stronger learning engagement among language learners (McEown et al., 2024). Stress has also been found to weaken the positive effects of psychological capital on academic engagement among postgraduate students (Saleem et al., 2022).

Although existing studies have linked academic pressure to motivation, engagement, anxiety, and foreign language enjoyment, the role of academic pressure in shaping foreign language boredom remains insufficiently examined. Given that boredom represents a deactivating achievement emotion closely associated with sustained academic demands, further investigation into this relationship is warranted.

### Control–value theory as an integrative framework

To integrate these constructs, the present study draws on Control–Value Theory (CVT) (Pekrun, 2006) of achievement emotions. According to CVT, learners’ emotions are primarily determined by two appraisals: perceived control over learning activities and perceived value of those activities. Positive emotions are more likely to emerge when learners experience high control and high value, whereas negative emotions such as boredom are associated with low perceived control, low value, or both (Pekrun, 2006; Pekrun et al., 2002; Pekrun and Perry, 2014).

Within AI-assisted academic English learning, AI literacy can be conceptualized as a key antecedent of perceived control, as it enables learners to understand, evaluate, and strategically use AI tools rather than passively depend on them. Higher AI literacy may therefore enhance learners’ sense of agency, which in turn promotes engagement and strengthens perceived task value. Engagement and task value, as proximal motivational variables, are expected to directly influence learners’ emotional experiences, including foreign language boredom. Academic pressure, meanwhile, may function as an additional emotional antecedent that intensifies boredom when learners perceive demands as overwhelming or misaligned with their control and value appraisals.

### Summary

In sum, existing literature suggests three critical points that shape the present research framework: (1) AI literacy is a developing but influential competence in educational and language learning contexts, with affective and psychological consequences; (2) engagement and task value are central motivational constructs that mediate learners’ experiences with technology-enhanced tasks; and (3) foreign language emotions, particularly boredom, have significant implications for learning outcomes, yet remain underexplored in AI-assisted academic English learning. These insights provide the foundation for examining how AI literacy, engagement, task value, and academic pressure interact to influence foreign language boredom among Chinese EFL postgraduates.

## Methodology

### Data collection

Data collection was conducted anonymously to ensure the confidentiality and privacy of participants. The study strictly adhered to the ethical guidelines of academic research integrity, and all necessary measures were taken to minimize potential risks and protect participants’ rights throughout the research process. Of the total 392 respondent postgraduate students, 309 (78.8%) students were male and 83(26.8%) were females and 325(82.9%) are majoring Engineering. Participants were recruited using Wenjuanxing Survey link, all participants were postgraduate students in a public engineering university in South China.

### Measures

#### AI literacy

AI literacy was measured using seven items adapted by Nong et al. (2024) to assess learners’ ability to understand, evaluate, and appropriately apply AI tools in academic English learning contexts. The items focused on students’ awareness of the functions and limitations of AI tools, their ability to evaluate the accuracy of AI-generated content, and their reflective use of AI feedback (e.g., “I can judge whether the English content or explanations provided by AI are correct”). Responses were rated on a Likert-type scale, with higher scores indicating higher levels of AI literacy. The scale demonstrated acceptable internal consistency in the present study (Cronbach’s *α* = 0.77).

#### AI learning engagement

AI learning engagement was assessed with six items adapted and reworded by Reeve and Tseng (2011) measuring learners’ emotional and cognitive engagement when using AI tools for English learning. These items captured students’ interest, sustained attention, and reflective thinking during AI-assisted learning activities (e.g., “When AI gives me feedback on my English, I carefully think about these suggestions”). Higher scores indicated greater engagement in AI-assisted English learning. The scale showed good reliability (Cronbach’s *α* = 0.85).

#### Perceived task value

Perceived task value was measured using four items adapted by Eccles (2005) assessing students’ perceptions of the usefulness and importance of academic English learning for their graduate studies and future research or professional development (e.g., “Improving my academic English ability is valuable for achieving my academic goals”). Higher scores reflected stronger perceived task value. The internal consistency of the scale was high (Cronbach’s α = 0.86).

#### Foreign language boredom

Foreign language boredom was measured with six items adapted by Li et al. (2023) capturing learners’ feelings of disengagement, monotony, and lack of interest during English learning activities (e.g., “I often feel bored when learning English”). All items were coded so that higher scores represented higher levels of boredom. The scale demonstrated excellent reliability in the present sample (Cronbach’s α = 0.90).

#### Academic pressure

Academic pressure was assessed using three items adapted by Misra and McKean (2000) measuring students perceived stress and emotional burden related to academic demands during their graduate studies (e.g., “The pressure of academic requirements makes me feel emotionally exhausted”). Higher scores indicated greater perceived academic pressure. The scale showed acceptable internal consistency (Cronbach’s α = 0.76).

The overall scale showed good internal consistency (Cronbach’s α = 0.87), while all subscales also demonstrated acceptable to excellent reliability. The internal consistency of all scales was examined using Cronbach’s alpha. As shown in Table 1, all constructs demonstrated satisfactory to excellent reliability, with Cronbach’s alpha coefficients ranging from 0.77 to 0.90. Specifically, the foreign language boredom scale showed excellent internal consistency (α = 0.90). Descriptive statistics indicated adequate variability across all constructs, suggesting that the data were suitable for subsequent factors and structural analyses.

**Table 1:** Descriptive statistics and reliability of the study variables (*N* = 392).

Construct	No. of items	M	SD	Cronbach’s *α*
AI literacy	7	27.49	4.16	0.77
AI learning engagement	6	23.96	3.94	0.85
Perceived task value	4	16.89	2.77	0.86
Foreign language boredom	6	19.08	5.88	0.90
Academic pressure	3	11.28	2.49	0.76
Overall scale	26	—	—	0.87

The perceived task value construct showed satisfactory reliability with Cronbach’s alpha of 0.86.

The academic pressure construct, consisting of three items, showed acceptable internal consistency (α = 0.76), which is considered adequate for short scales.

## Data analysis

### Preliminary analyses

#### Common method Bias

Because all variables were measured using self-report questionnaires collected at a single time point, common method variance (CMV) was assessed using Harman’s Single-factor Test. An unrotated principal component analysis including all measurement items showed that the first factor accounted for 34.61% of the total variance, below the commonly used 40% threshold. This suggests that CMV was unlikely to substantially distort the observed associations, although method bias cannot be completely ruled out.

Prior to conducting exploratory factor analysis (EFA), the suitability of the data was assessed. The Kaiser–Meyer–Olkin (KMO) measure of sampling adequacy was 0.901, indicating excellent sampling adequacy. Bartlett’s test of Sphericity was significant, χ^2^(325) = 5495.64, *p* < 0.001, suggesting that the correlation matrix was appropriate for factor analysis.

#### Exploratory factor analysis

Exploratory factor analysis (EFA) was conducted using principal axis factoring with Promax rotation. The Kaiser–Meyer–Olkin measure of sampling adequacy was 0.901, and Bartlett’s test of Sphericity was significant (χ^2^ = 5495.64, df = 325, *p* < 0.001), indicating that the data were suitable for factor analysis.

Four factors with eigenvalues greater than 1 were extracted, accounting for 59.67% of the total variance. As shown in Table 2, all items loaded primarily with their intended factors, with factor loadings ranging from 0.46 to 0.85, and no problematic cross-loadings were observed.

**Table 2:** Exploratory factor analysis results (PAF with Promax rotation).

Item	Foreign language boredom	AI literacy	Perceived task value	Academic pressure	AI-assisted learning engagement
I feel mentally disengaged when studying English.	0.849				
English learning tasks often fail to hold my attention.	0.803				
English learning activities feel repetitive and uninteresting.	0.799				
I feel bored during English learning activities.	0.791				
I struggle to stay focused during English classes or tasks.	0.771				
Time passes slowly when I am doing English learning tasks.	0.637				
I avoid relying excessively on AI when completing English learning tasks.		0.745			
I can judge whether AI-generated English texts or explanations are accurate.		0.644			
I understand what AI tools can and cannot do when supporting my English learning.		0.518			
I know how to use AI tools effectively for academic English tasks (e.g., reading, writing, revising).		0.484			
I am aware of the limitations of AI-generated English content.		0.461			
AI-supported English learning makes me feel emotionally involved.					0.676
I actively reflect on how AI suggestions can improve my English learning.					0.623
I invest effort in understanding AI-assisted English learning materials.					0.604
I enjoy engaging with English tasks that involve AI tools.					0.554
I try to think deeply when using AI feedback to improve my English.					0.546
English learning tasks are useful for my future research or career.			0.793		
Improving my academic English skills is valuable for achieving my academic goals.			0.732		
English learning activities are meaningful for my postgraduate studies.			0.689		
Learning English is important for my postgraduate academic development.			0.551		
Academic expectations make my postgraduate studies emotionally demanding.				0.631	
I feel strong pressure to meet academic requirements in my postgraduate program.				0.560	
I feel pressure to improve my English for academic purposes (e.g., publishing, presentations).				0.527	

Factor loadings below 0.40 are suppressed. Extraction method: principal axis factoring. Rotation method: Promax with Kaiser normalization.

#### Confirmatory factor analysis

A CFA was conducted to evaluate the hypothesized five-factor measurement model (AI literacy, learner engagement, perceived task value, academic pressure, and foreign language boredom). The model demonstrated acceptable-to-marginal fit. Model fit indices are presented in Table 3.

**Table 3:** CFA model fit indices.

Fit index	Value	Criterion	Evaluation
χ^2^/df	3.02	<5	Acceptable
CFI	0.830	≥0.90 preferred	Marginal
TLI	0.818	≥0.90 preferred	Marginal
RMSEA	0.072	≤0.08	Acceptable

The results indicated that the model demonstrated an acceptable overall fit to the data. Specifically, the chi-square to degrees of freedom ratio (χ^2^/df) was 3.02, which falls below the recommended threshold of 5, indicating an acceptable level of model fit. The root mean square error of approximation (RMSEA) was 0.072, which is below the commonly accepted cutoff value of 0.08, suggesting reasonable approximation error.

However, the comparative fit index (CFI = 0.830) and Tucker–Lewis index (TLI = 0.818) were slightly below the preferred criterion of 0.90, indicating marginal incremental fit. Although these values did not reach the ideal threshold, they approached acceptable levels and may be considered tolerable in complex multi-factor models with numerous observed indicators.

Taking together, the fit indices suggest that the five-factor measurement model provides an adequate, though not optimal, representation of the data. Given the theoretical grounding of the constructs and the acceptable absolute fit indices (χ^2^/df and RMSEA), the measurement model was retained for subsequent structural analyses.

### Structural equation model

See Figure 1.

**Figure 1:**
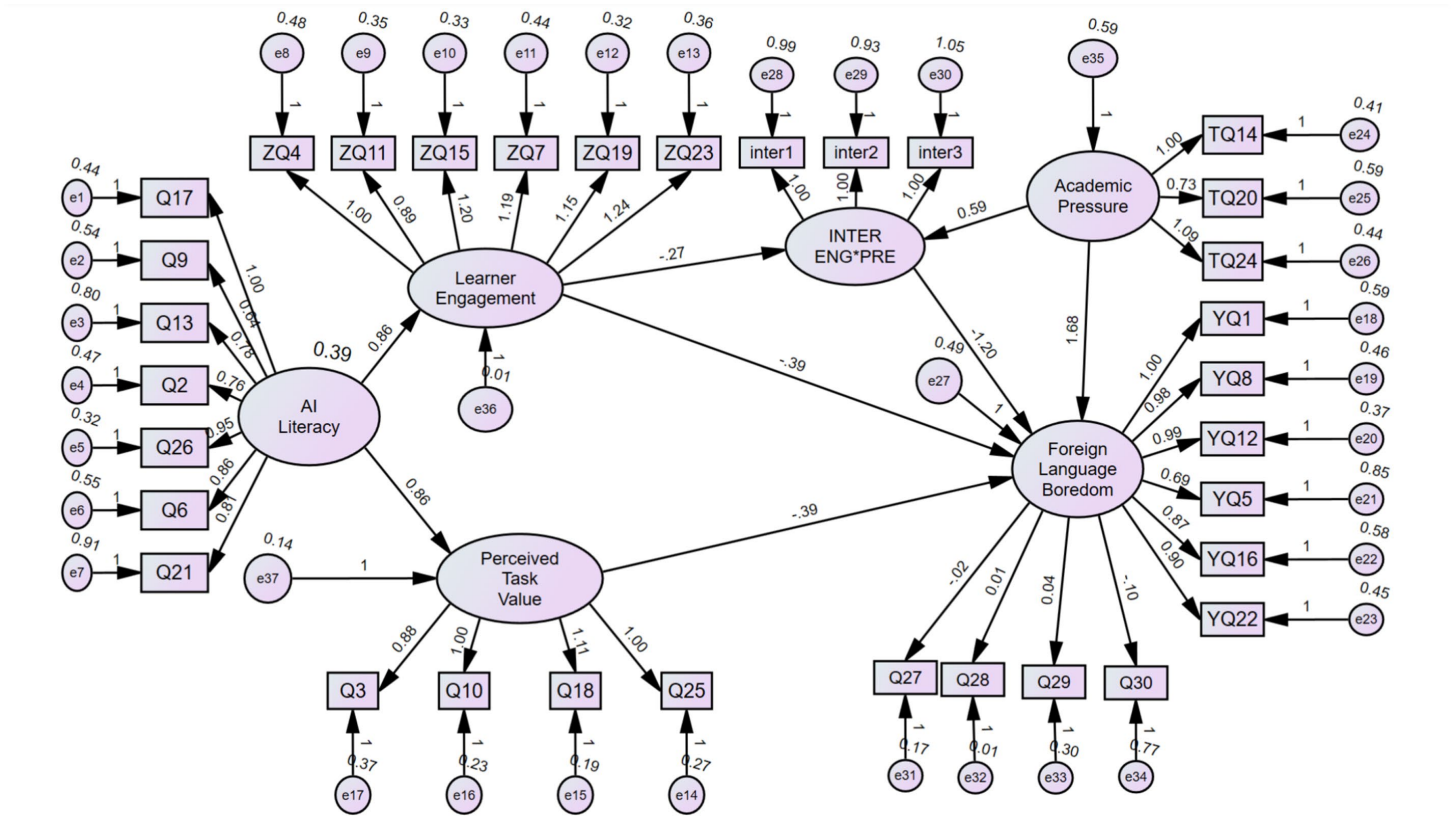
Structural equation model.

### Model fit indices

To evaluate the overall fit of the structural equation model, multiple commonly used fit indices were employed, including the chi-square to degrees of freedom ratio (χ^2^/df), the goodness-of-fit index (GFI), the comparative fit index (CFI), the Tucker–Lewis index (TLI), the root mean square error of approximation (RMSEA), and the Hoelter critical N. The results of these fit indices are presented in Table 4.

**Table 4:** Model fit indices and evaluation criteria.

Fit index	Value	Recommended criterion	Model fit evaluation
χ^2^/df	3.201	< 5	Acceptable
GFI	0.795	> 0.80	Marginally acceptable
CFI	0.815	> 0.80	Acceptable
TLI	0.802	> 0.80	Acceptable
RMSEA	0.075	< 0.08	Reasonable fit
HOELTER (0.05)	136	> 100	Acceptable

The overall model fit was evaluated using multiple absolute and incremental fit indices. Regarding absolute fit indices, the chi-square to degrees of freedom ratio (χ^2^/df = 3.20) was below the recommended threshold of 5, indicating an acceptable balance between model complexity and the observed data. In addition, the root mean square error of approximation (RMSEA = 0.075) was below the commonly accepted cutoff of 0.08, suggesting a reasonable level of approximation error and an acceptable overall model fit.

With respect to incremental fit indices, the comparative fit index (CFI = 0.815) and Tucker–Lewis index (TLI = 0.802) both exceeded the minimum recommended criterion of 0.80. These results indicate that, compared with the independence model, the proposed structural model provided a meaningful improvement in explaining the covariance structure of the data. Although these values did not reach the more stringent 0.90 benchmark, they are considered statistically acceptable given the relative complexity of the model and the exploratory nature of the research.

The goodness-of-fit index (GFI = 0.795) was slightly below the conventional cutoff of 0.80, suggesting that there remains some room for improvement in explaining the sample covariance matrix. However, as GFI is known to be sensitive to sample size and model complexity, its influence on the overall evaluation of model fit was considered limited when interpreted alongside other fit indices.

In terms of sample size adequacy, the Hoelter critical N at the 0.05 significance level was 136, exceeding the recommended minimum of 100. This result indicates that the current sample size was sufficient to support stable estimation of the proposed model.

Although the overall model demonstrated acceptable fit, it is worth noting that the GFI value was slightly below the conventional threshold. One possible explanation lies in the complexity of the proposed model, which included multiple interrelated latent constructs and structural paths. Previous methodological research has indicated that GFI is particularly sensitive to model complexity and sample size. In models involving several psychological constructs, minor deviations from recommended cutoffs are not uncommon. Therefore, the slightly lower GFI should be interpreted cautiously and in conjunction with other fit indices, which collectively suggested an acceptable and stable model fit.

Taken together, the fit indices generally met or closely approached recommended thresholds, suggesting that the proposed structural model demonstrated an acceptable overall fit and was suitable for subsequent path coefficient estimation.

### Structural path analysis

To test the proposed hypotheses, structural equation modeling was employed to estimate the relationships among the latent variables. Table 5 presents the unstandardized estimates, standard errors, critical ratios, significance levels, and standardized path coefficients.

**Table 5:** Path coefficients of the structural equation model.

Path	Estimate	S.E.	C.R.	*p*	Standardized β
Learning Engagement←AI Literacy	0.857	0.063	13.662	***	0.984
Task Value←AI Literacy	0.814	0.053	15.462	***	0.816
Foreign Language Boredom←Learning Engagement	−0.395	0.139	2.841	0.005	−0.395
Foreign Language Boredom←Task Value	0.257	0.124	2.067	0.039	0.257
Academic Pressure←Learning Engagement	−0.309	0.089	11.529	***	−0.309
Foreign Laguage Boredom←Academic Pressure	1.204	0.092	13.067	***	0.951
Foreign Language Boredom←Engagement x Task Value	−1.204	0.276	1.067	0.286	−0.12

As expected, AI literacy showed a strong and significant positive effect on learning engagement (*β* = 0.984, *p* < 0.001), indicating that students with higher levels of AI literacy were more actively engaged in AI-assisted English learning. AI literacy also significantly associated with perceived task value (β = 0.816, *p* < 0.001), suggesting that greater competence in understanding and using AI tools was associated with higher perceptions of the value and usefulness of English learning tasks. These findings support AI literacy as a key antecedent of both motivational and behavioral learning processes.

Learning engagement demonstrated a significant negative association with foreign language boredom (β = −0.395, *p* = 0.005), indicating that higher engagement was associated with lower levels of boredom in English learning. In addition, learning engagement negatively associated with academic pressure (β = −0.309, *p* < 0.001), suggesting that engaged learners experienced lower levels of perceived academic strain.

Perceived task value showed a small but significant positive relationship with foreign language boredom (β = 0.257, *p* = 0.039). This result suggests that, in the context of postgraduate-level academic English learning, perceiving tasks as highly valuable may also be accompanied by increased cognitive or emotional demands, which in turn could contribute to feelings of boredom.

Academic pressure emerged as a strong positive predictor of foreign language boredom (β = 0.951, *p* < 0.001), indicating that students experiencing higher levels of academic pressure were substantially more likely to report boredom in English learning activities. This finding highlights academic pressure as a central emotional antecedent of foreign language boredom.

Finally, the interaction relationship between learning engagement and perceived task value on foreign language boredom was not statistically significant (β = −0.12, *p* = 0.286), suggesting that perceived task value did not moderate the relationship between learning engagement and boredom.

### Mediation analysis

To further examine the indirect pathways through which AI literacy influences foreign language boredom, a bootstrapping procedure was conducted to test the proposed mediation effects. Bias-corrected bootstrap confidence intervals (95% CI) were generated based on 5,000 resamples. The results are presented in Table 6.

**Table 6:** Mediation analysis results.

Path	Indirect effect	BootS.E.	95%CI (lower)	95%CI (upper)
AI Literacy→Engagement→Foreign Language Boredom	−0.184	0.046	−0.28	−0.09
AI Literacy→Task Value→Foreign Language Boredom	−0.034	0.017	−0.06	−0.01
AI Literacy→Engagement→Academic Pressure→Foreign Language Boredom	−0.026	0.022	−0.08	0.01

First, the indirect association of AI literacy on foreign language boredom via learner engagement was statistically significant (indirect association = −0.184, Boot SE = 0.046, 95% CI [−0.28, −0.09]). Because the confidence interval did not include zero, this finding indicates that learner engagement significantly mediated the relationship between AI literacy and foreign language boredom. Specifically, higher levels of AI literacy were associated with greater learner engagement, which in turn related to lower levels of foreign language boredom.

Second, perceived task value also served as a significant mediator in the relationship between AI literacy and foreign language boredom. The indirect association through task value was negative and statistically significant (indirect association = −0.034, Boot SE = 0.017, 95% CI [−0.06, −0.01]), suggesting that AI literacy reduced foreign language boredom by enhancing learners’ perceptions of task value in AI-assisted academic English learning.

In contrast, the serial mediation pathways from AI literacy to foreign language boredom through learner engagement and academic pressure was not supported. Although the indirect association was negative in direction (indirect association = −0.026, Boot SE = 0.022), the corresponding 95% bootstrap confidence interval included zero [−0.08, 0.01], indicating although engagement was negatively associated with academic pressure, the combined indirect pathway was insufficient to produce a reliable serial mediation association.

Overall, these findings suggest that learner engagement and perceived task value independently function as significant mediators linking AI literacy to foreign language boredom, whereas the serial mediation through academic pressure was not empirically supported.

## Discussion

This study examined the structural relationships among AI literacy, learning engagement, perceived task value, academic pressure, and foreign language boredom in the context of AI-assisted academic English learning among Chinese postgraduate students. Although the structural model demonstrated an acceptable level of fit, several indices (e.g., GFI, TLI) were slightly below conventional thresholds. This may be related to the complexity of the model, which includes multiple latent constructs and mediating paths. Previous SEM studies have noted that model fit indices can be sensitive to model complexity and sample characteristics. Therefore, the findings should be interpreted as providing tentative rather than definitive evidence for the proposed relationships. Overall, the findings provide empirical support for the proposed model and offer several theoretical and practical insights into how AI-related competencies shape learners’ motivational, emotional, and behavioral experiences.

### The central role of AI literacy in AI-assisted language learning

One of the most prominent findings of this study is the strong predictive role of AI literacy in both learning engagement and perceived task value. AI literacy demonstrated a substantial positive effect on learning engagement (*β* = 0.984), suggesting that students who possess a clearer understanding of the capabilities, limitations, and appropriate use of AI tools are more likely to invest cognitive, behavioral, and emotional resources in AI-assisted English learning. This finding aligns with prior research emphasizing that technological competence and metacognitive awareness are critical prerequisites for effective technology-enhanced learning (Wang et al., 2025).

Similarly, AI literacy significantly enhanced students’ perceptions of task value (β = 0.816). When learners are able to evaluate AI-generated content, use AI tools strategically, and maintain awareness of ethical boundaries, they are more likely to view English learning tasks as meaningful, useful, and relevant to their academic goals. Taken together, these results position AI literacy as a foundational antecedent that shapes both motivational appraisals and engagement behaviors in AI-supported learning environments. This extends existing models of digital literacy by demonstrating its downstream emotional and motivational consequences in foreign language learning contexts.

### Learning engagement as a protective factor against negative academic emotions

Consistent with expectations, learning engagement played a protective role by reducing both foreign language boredom (β = −0.395) and academic pressure (β = −0.309). Students who were more engaged in AI-assisted English learning reported lower levels of boredom, indicating that active involvement, sustained attention, and reflective use of AI feedback can mitigate monotonous or disengaging learning experiences. This aligns with the study that emotions like boredom affect academic engagement and achievement (Borgonovi et al., 2023). This finding supports engagement-based models of emotion regulation, which emphasize that meaningful participation can buffer against negative affective states in academic settings.

Moreover, learning engagement was negatively associated with academic pressure (*β* = −0.309). This suggests that engagement may function not only as a motivational outcome but also as a coping pathway, helping learners manage the demands of graduate-level academic English tasks. Engaged learners may perceive academic challenges as more controllable and less threatening, thereby experiencing lower levels of stress and emotional exhaustion. These findings reinforce the importance of fostering engagement as a key pathway for improving both emotional well-being and learning sustainability.

### The complex role of perceived task value in foreign language boredom

Interestingly, perceived task value showed a small but significant positive relationship with foreign language boredom (β = 0.257). Although this finding may appear counterintuitive, as it does not align with the previous study which found negative association between task value and boredom (Sánchez-Rosas and Esquivel, 2016). However, this finding reflects the complex emotional dynamics of graduate-level academic learning in China. In high-stakes academic contexts, tasks perceived as highly valuable, such as those related to thesis writing, publication, or academic presentations, may also entail increased cognitive load, performance pressure, and prolonged effort. As a result, learners may simultaneously recognize the importance of English learning tasks while experiencing boredom or emotional fatigue during task execution. Under such conditions, the perceived importance of academic tasks may intensify learners’ psychological burden, which can paradoxically contribute to feelings of boredom when cognitive resources are strained or when learners experience fatigue from sustained academic demands. Furthermore, in AI-assisted learning environments, highly valued tasks may involve extensive interaction with AI tools, critical evaluation of AI-generated outputs, and repeated revisions of academic texts. While these activities are academically meaningful, they may also increase cognitive load and prolong task engagement, potentially leading to emotional exhaustion or disengagement over time. Therefore, in high-pressure academic contexts, perceived task value may not always function as a purely motivational resource but may also interact with academic stressors in ways that contribute to boredom.

This finding suggests that high task value does not automatically guarantee positive emotional experiences. Instead, when value perceptions are not accompanied by sufficient autonomy, variety, or emotional support, learners may still experience boredom. This nuanced result contributes to the literature by highlighting that task value and learning emotions can coexist in complex and sometimes contradictory ways, particularly in advanced academic contexts.

### Academic pressure as a dominant predictor of foreign language boredom

Academic pressure emerged as the strongest predictor of foreign language boredom in the model (*β* = 0.951). This result underscores that boredom in academic English learning is not merely a function of task design or individual motivation but is deeply embedded in broader academic stressors (Klimova and Pikhart, 2025; Saleem et al., 2022). High expectations related to publication, academic performance, and future career development may deplete learners’ emotional resources, making them more susceptible to boredom even when tasks are meaningful or well-structured.

This finding extends previous research on foreign language emotions by emphasizing the role of contextual pressure in shaping learners’ affective experiences. It suggests that interventions aimed at reducing boredom should not focus solely on instructional strategies but also address systemic academic pressures that influence learners’ emotional states.

### Absence of a moderating association of perceived task value

Contrary to expectations, the interaction association between learning engagement and perceived task value on foreign language boredom was not statistically significant (β = −0.12). This indicates that perceived task value did not moderate the relationship between engagement and boredom. One possible explanation is that engagement exerts a relatively stable relationship with boredom regardless of learners’ value perceptions. Alternatively, in highly demanding academic contexts, the emotional impact of engagement may be overshadowed by broader stress-related factors, such as academic pressure.

The absence of a significant moderation association does not undermine the overall model but rather suggests that the relationship between engagement and boredom is robust across different levels of perceived task value. Future research may explore alternative moderators, such as emotional regulation strategies or perceived autonomy, to further clarify boundary conditions.

## Conclusion

This study examined the relationships among AI literacy, learning engagement, perceived task value, academic pressure, and foreign language boredom in AI-assisted academic English learning among postgraduate students. Several key conclusions can be drawn.

First, AI literacy is strongly associated with learners’ motivational processes in AI-supported learning environments. Students with higher levels of AI literacy demonstrated substantially stronger learning engagement and perceived greater task value, suggesting that technological competence plays a critical role in enabling meaningful participation in AI-assisted language learning.

Second, learning engagement functioned as an important protective factor in learners’ emotional experiences. Higher engagement was associated with lower levels of both academic pressure and foreign language boredom, indicating that active cognitive and behavioral involvement in learning activities may help buffer negative emotions in demanding academic contexts.

Third, academic pressure was identified as the strongest predictor of foreign language boredom. This finding highlights that boredom in academic English learning is not merely a result of task characteristics or motivational deficits but is also strongly influenced by broader academic stressors faced by graduate students.

Finally, the findings reveal a chain pathway linking technological competence to emotional outcomes. Specifically, AI literacy promotes learning engagement and perceived task value, which subsequently influences learners’ perceptions of academic pressure and ultimately shapes their experience of foreign language boredom. This pathway underscores the interconnected roles of technological skills, motivational processes, and contextual pressures in shaping learners’ emotional experiences in AI-mediated learning environments.

Taken together, the present study contributes to a growing body of research on emotions in technology-enhanced language learning by demonstrating how AI literacy may indirectly associate with learners’ emotional outcomes through motivational and contextual pathways. As artificial intelligence becomes increasingly integrated into higher education, understanding these pathways will be essential for designing learning environments that foster both effective engagement and positive emotional experiences.

## Implications

### Theoretical implications

This study offers several theoretical contributions to literature on foreign language learning and AI-assisted education. First, it extends existing models of foreign language emotions by integrating AI literacy as a core antecedent variable. While previous research has primarily focused on general digital literacy or technology acceptance, the present findings demonstrate that AI-specific literacy has distinct motivational and emotional consequences in language learning contexts.

Second, the study enriches engagement-based frameworks by positioning learning engagement as both an outcome of AI literacy and a mediator influencing emotional variables such as boredom and academic pressure. This highlights engagement as a central linking technological competence with emotional regulation in academic learning.

Third, the positive association between perceived task value and foreign language boredom challenges the assumption that higher task value uniformly leads to positive emotional outcomes. This finding suggests that, particularly in high-stakes academic settings, task value may coexist with emotional exhaustion or boredom, calling for more nuanced theoretical models that account for the emotional costs of highly valued learning tasks.

### Practical implications

The findings of this study provide several practical implications for improving the emotional and motivational dynamics of AI-assisted academic English learning. These implications can be considered at three interconnected levels: teachers, learners, and educational institutions.

At the teacher level, the results highlight the importance of integrating AI literacy into instructional design. Since AI literacy demonstrated a strong influence on both learning engagement and perceived task value, instructors should move beyond simply allowing the use of AI tools and instead provide explicit guidance on how to use them critically and strategically. For example, teachers may design activities that require students to evaluate AI-generated feedback, compare AI-generated texts with human-authored models, or reflect on the strengths and limitations of AI assistance. Such practices can promote deeper cognitive engagement and help students develop a more informed and purposeful approach to AI-supported language learning.

At the learner level, the findings suggest that developing students’ self-regulated learning strategies when using AI tools may help mitigate negative emotional experiences such as boredom. Students who actively engage with AI-generated feedback, revise their work iteratively, and critically assess AI output may experience greater involvement in the learning process, which can reduce disengagement. Encouraging reflective use of AI, rather than passive reliance on automated responses, may therefore enhance both learning engagement and perceived task meaningfulness.

At the institutional level, the strong association between academic pressure and foreign language boredom indicates that emotional experiences in academic English learning are shaped not only by instructional factors but also by broader academic environments. Universities may therefore consider providing structural support systems that help graduate students manage academic demands more effectively. These may include workshops on academic writing supported by AI tools, training programs on responsible AI use in research and learning, and counseling or mentoring services aimed at reducing excessive academic stress. By fostering a supportive academic environment and promoting responsible AI literacy, institutions can help create conditions that sustain both learner engagement and emotional well-being in AI-assisted learning contexts.

### Limitations

Despite its contributions, this study has several limitations that should be acknowledged. First, the data were collected using self-report questionnaires, which may be subject to common method bias and social desirability effects. Although the measurement model demonstrated acceptable reliability and validity, future studies could incorporate behavioral data or qualitative evidence to triangulate the findings. In addition, as the data were cross-sectional, the findings should be interpreted as statistical associations rather than evidence of causal relationships.

Second, the cross-sectional research design limits causal interpretations of the relationships among variables. While the structural model is theoretically grounded, longitudinal or experimental designs are needed to more rigorously examine causal pathways.

Third, the sample consisted of graduate-level learners within a specific educational context, which may limit the generalizability of the findings. Learners at different educational levels or from different cultural or institutional backgrounds may experience AI-assisted language learning differently.

### Future directions

Building on the current findings, several directions for future research are suggested. First, future studies could adopt longitudinal designs to examine how AI literacy, engagement, and emotional variables evolve over time as learners gain more experience with AI tools.

Second, additional affective variables such as enjoyment, anxiety, or emotional regulation strategies could be incorporated to develop a more comprehensive emotional framework for AI-assisted foreign language learning.

Third, future research could explore alternative moderating variables, such as perceived autonomy, instructional design features, or teacher support, to better understand the boundary conditions under which engagement and task value influence foreign language boredom.

Fourth, future research may also explore the moderating role of different AI tool types (e.g., generative AI vs. adaptive learning systems) in shaping learners’ engagement and emotional experiences. Additionally, intervention studies could investigate whether AI literacy training programs effectively enhance engagement and reduce negative emotions in AI-assisted language learning contexts.

Finally, mixed-methods approaches combining quantitative modeling with qualitative interviews or learning analytics could provide richer insights into EFL students lived experiences and the pathways underlying their emotional responses to AI-assisted learning.

## Data Availability

The raw data supporting the conclusions of this article will be made available by the authors, without undue reservation.
